# Supplementary material for: Identification of photoperiod-induced specific miRNAs in the adrenal glands of Sunite sheep (Ovis aries)
Source: Front Vet Sci. 2022 Jul 22;9:888207. doi: 10.3389/fvets.2022.888207 (PMC9354845; doi:10.3389/fvets.2022.888207)
Supplement: Supplementary file 1 [file Data_Sheet_1.ZIP › Supplementary file/miRDeep2 file.zip!/miRDeep2 file/Know_Structure/LP42A3/oar-mir-369.pdf]

miRBase precursor : oar-mir-369
Total read count : 21275
oar-miR-369-5p read count : 1755
oar-miR-369-3p read count : 19518
remaining reads : 2

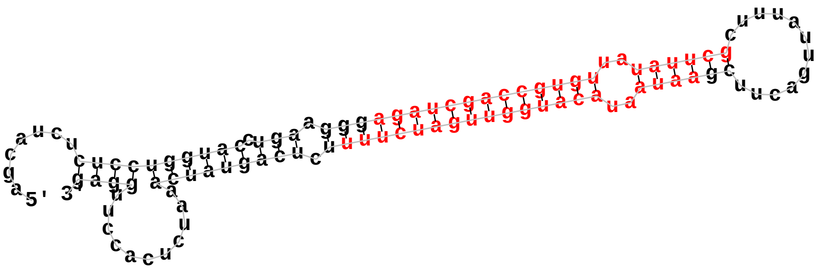

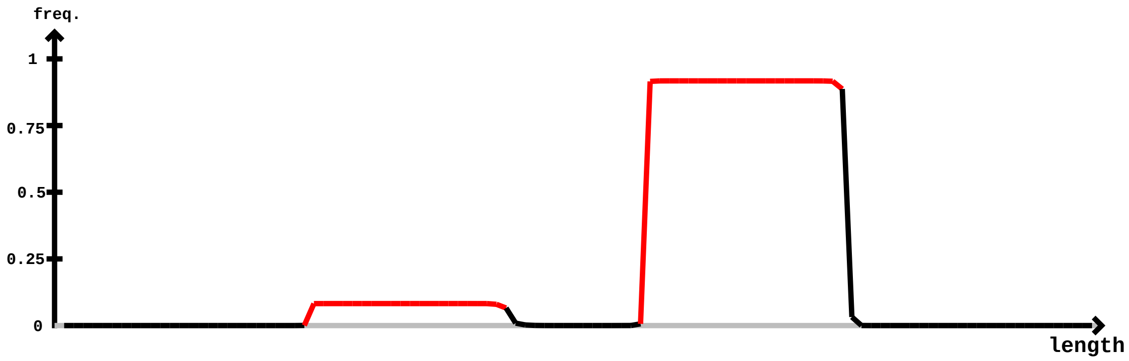

oar-miR-369-5p

oar-miR-369-3p

```
5'- agcaucucuccugguaccugaagggagaucgaccguguuauauucgcuuuauugacuucgaauaauacaugguugaucuuuucucaguaucaaaucucaccuuggag   -3'    exp
    .......((((((((((.(((.((((((((((((((((..((((((............))))))..)))))))))))))))).)))))))))...........))))   reads   mm   sample
    .......................gagaucgaccguguuauauu................................................................   1       0    seq
    .......................gagaucgaccguguuauauuc...............................................................   1       0    seq
    .......................gagaucgaccguguuauauucg..............................................................   8       0    seq
    .........................agaucgaccguguuaua.................................................................   1       0    seq
    .........................agaucgaccguguuauauu...............................................................   57      0    seq
    .........................agaucgaccguguuauauuc..............................................................   293     0    seq
    .........................agaucgaccguguuauauucg.............................................................   1198    0    seq
    .........................agaucgaccguguuauauucgc............................................................   144     0    seq
    .........................agaucgaccguguuauauucgcu...........................................................   38      0    seq
    .........................agaucgaccguguuauauucgcuu..........................................................   9       0    seq
    ..........................gaucgaccguguuauauucg.............................................................   2       0    seq
    ...........................aucgaccguguuauauucg.............................................................   1       0    seq
    ...........................aucgaccguguuauauucgcuuu.........................................................   2       0    seq
    .........................................................ucgaauaauacaugguugaucuu...........................   1       0    seq
    .........................................................ucgaauaauacaugguugaucuuu..........................   1       0    seq
    ..........................................................cgaauaauacaugguugaucuuu..........................   3       0    seq
    ...........................................................gaauaauacaugguugaucuu...........................   19      0    seq
    ...........................................................gaauaauacaugguugaucuuu..........................   113     0    seq
    ...........................................................gaauaauacaugguugaucuuuu.........................   1       0    seq
    ............................................................aauaauacaugguugauc.............................   5       0    seq
    ............................................................aauaauacaugguugaucu............................   26      0    seq
    ............................................................aauaauacaugguugaucuu...........................   595     0    seq
    ............................................................aauaauaAaugguugaucuu...........................   1       1    seq
    ............................................................aauaauacaugguugaucuuu..........................   18044   0    seq
    ............................................................aauaauaAaugguugaucuuu..........................   2       1    seq
    ............................................................aauaauacaugguugaucuuuu.........................   665     0    seq
    ............................................................aauaauacaugguugaucuuuuc........................   1       0    seq
    .............................................................auaauacaugguugaucuu...........................   1       0    seq
    .............................................................auaauacaugguugaucuuu..........................   23      0    seq
    .............................................................auaauacaugguugaucuuuu.........................   14      0    seq
    .............................................................auaauacaugguugaucuuuuc........................   2       0    seq
    ..............................................................uaauacaugguugaucuuu..........................   1       0    seq
    ..............................................................uaauacaugguugaucuuuucuc......................   1       0    seq
    ...............................................................aauacaugguugaucuuu..........................   1       0    seq
```

oar-miR-369-5p

oar-miR-369-3p

agcaucucuccugguaccugaagggagaucgaccguguuauauucgcuuuauugacuucgaauaauacaugguugaucuuuucucaguaucaaaucucaccuuggag
